# Disability and quality of life 20 years after traumatic brain injury

**DOI:** 10.1002/brb3.1018

**Published:** 2018-06-11

**Authors:** Nada Andelic, Emilie I. Howe, Torgeir Hellstrøm, Maria Fernandez Sanchez, Juan Lu, Marianne Løvstad, Cecilie Røe

**Affiliations:** ^1^ Department of Physical Medicine and Rehabilitation Oslo University Hospital Ulleval Norway; ^2^ Institute of Health and Society Research Centre for Habilitation and Rehabilitation Models and Services (CHARM) Faculty of Medicine University of Oslo Oslo Norway; ^3^ Institute of Clinical Medicine Faculty of Medicine University of Oslo Oslo Norway; ^4^ Institute on Community Integration (INICO) Faculty of Psychology University of Salamanca Salamanca Spain; ^5^ Division of Epidemiology Department of Family Medicine and Population Health Virginia Commonwealth University Richmond Virginia; ^6^ Department of Research Sunnaas Rehabilitation Hospital Nesoddtangen Norway; ^7^ Department of Psychology University of Oslo Oslo Norway

**Keywords:** functional outcomes, long‐term follow‐up, quality of life, traumatic brain injury

## Abstract

**Objectives:**

The study describes functional outcomes and health‐related quality of life (HRQL) in patients with traumatic brain injury (TBI) 20 years postinjury.

**Materials and Methods:**

Forty‐four survivors who acquired moderate and severe TBI during 1995–1996 were followed 10 and 20 years postinjury. Outcomes were Glasgow Outcome Scale Extended (GOSE), Community Integration Questionnaire (CIQ), and SF‐36 questionnaire (SF‐36). Multiple regressions were performed to examine the relationship between follow‐up measurements, controlling for baseline demographics and injury severity.

**Results:**

There were no significant differences in baseline age and civil status between moderate and severe TBI, but patients with severe injury had significantly lower employment rates (*p* = 0.05). Mean age at 20‐years follow‐up was 50.8 (*SD* 11.4) years, and 73% were males. Most patients showed good recovery (52%) or moderate disability (43%). Disability levels remained stable between and within severity groups from 10 to 20 years. Community integration including social integration improved from 10 to 20 years (*p* = 0.01 and *p* = 0.005, respectively). HRQL remained stable, except for subscales Bodily Pain and Role Emotional (*p* = 0.02 and *p* = 0.06). Depression at 10 years and females were associated with poorer mental health, while productive activity at 10 years indicated better physical and mental health at 20 years postinjury, respectively.

**Conclusions:**

Functional limitations persist even decades after moderate and severe TBI, with poorer prognosis for females and persons who were depressed at the 10‐year follow‐up. Development and evaluation of targeted long‐term follow‐up programs and access to rehabilitation services for these groups should be highlighted. Improved community integration despite stable functional limitations draws attention to long‐term adaptation to adversity and illness.

## 1 INTRODUCTION

A large proportion of patients with moderate and severe traumatic brain injury (TBI) sustain long‐term physical, cognitive, and emotional impairments that have a deep impact on their functioning, reintegration to society and health‐related quality of life (HRQL; Andelic et al., 2009; Forslund, Roe, Sigurdardottir, & Andelic, 2013; Hammond et al., 2004; Jacobsson, Westerberg, Soderberg, & Lexell, 2009; Ponsford, Draper, & Schonberger, 2008; Schulz‐Heik et al., 2016; Sigurdardottir, Andelic, Roe, & Schanke, 2009; Wilson et al., 2017). TBI outcomes up to 10 years postinjury have been documented in several studies (Andelic et al., 2009, 2015; Dahm & Ponsford, 2015; Jourdan et al., 2016). The findings suggest that patients with moderate and severe TBI experience persisting functional limitations and decreased employment rates (Andelic et al., 2009; Forslund et al., 2017; Ponsford et al., 2008), reduced physical and mental health and elevated probabilities of social isolation (Andelic et al., 2009; Hawthorne, Gruen, & Kaye, 2009). Being older and female contribute to decreased physical and mental health (Andelic et al., 2009; Forslund et al., 2013), whereas being employed or involved in productive activities 10 years after injury contributes to better physical functioning, social and psychological well‐being and HRQL (Andelic et al., 2009; Ponsford et al., 2008). Taken together, the studies indicate that persons with TBI may need professional assistance to improve functioning and quality of life, even a decade postinjury.

There is limited research worldwide on the very long‐term outcomes after TBI (up to 20 years postinjury; Brown et al., 2011; Hoofien, Gilboa, Vakil, & Donovick, 2001; Nestvold & Stavem, 2009; Steadman‐Pare, Colantonio, Ratcliff, Chase, & Vernich, 2001), and few studies have been conducted in Europe. Among these, McMillan, Teasdale, and Stewart (2012) found that half of the young people and adults that were assessed reported disability 12–14 years after head injury. Lexell, Wihlney, and Jacobsson (2016) assessed disability 6–15 years after injury and confirmed a strong association between disability and occupational status. Wood and Rutterford (Wood & Rutterford, 2006) assessed outcomes on average 17 years after severe injury and reported that although long‐term psychosocial functioning remains weakened, community integration levels were just below those reported for nondisabled patients. Jacobsson, Westerberg, & Lexell (2010) evaluated HRQL and life satisfaction 6–15 years after TBI. Compared to a reference sample, the TBI patients reported lower HRQL, except for subscale RE (role emotional) and the mental component summary (MCS). Patients who were injured a longer time ago reported better overall health compared to those injured more recently. Furthermore, Nestvold & Stavem assessed determinants of HRQoL in a TBI cohort 22 years after injury, and found no association between HRQoL and injury data (Nestvold & Stavem, 2009).

It is challenging to establish which long‐term services are needed to target functional domains that patients with TBI find particularly difficult (Hoofien et al., 2001), and not only HRQL as has been performed in previous studies. Multidimensional functional assessments may help patients and care providers define common goals, and coordinate service delivery. Such studies are important both from the epidemiological and rehabilitative perspectives (Hoofien et al., 2001). The present study aims to broaden the knowledge base on the functional outcomes and factors that contribute in a long‐term perspective by following a patient cohort with moderate and severe TBI from 10 to 20 years after injury. The main objective was to describe disability levels and HRQL 20 years after injury. Second, we studied changes in global functioning, community integration and HRQL from 10 to 20 years postinjury, and identified factors associated with physical and mental health at 20‐year follow‐up. Finally, HRQL in the study population was compared to the general Norwegian population.

## 2 MATERIALS AND METHODS

The study includes a 20‐year follow‐up of a 2‐year TBI cohort injured in 1995/1996, consisting of 62 patients with moderate and severe TBI who were admitted to the university‐affiliated Trauma Referral Center in Oslo, Norway.

The Glasgow Coma Scale score (GCS; Teasdale & Jennett, 1974) at the time of emergency admission to the hospital was used to classify injury severity (moderate injury 9–12 vs. severe 3–8). Baseline data, including socio‐demographics and injury‐related factors, were extracted from the hospital's medical records at the 10‐year follow‐up which was completed in 2005/2006, and reported previously (Andelic et al., 2009).

The 20‐year follow‐up was performed in 2015/2016. The Regional Committee for Medical Research Ethics, South‐East Norway approved the study (2015/389). Written informed consent was obtained. Participants were contacted by mail and/or telephone, thereafter a face‐to‐face interview with physiatrists (NA and TH, *n* = 27) was scheduled. When a direct interview was not possible, patients or their relatives were contacted by telephone (EIH and NA, *n* = 17) to obtain information, including socio‐demographics such as living situation, employment and outcome data, that is, functional status, community integration and HRQL.

### 2.1 Outcome measures applied at 10‐ and 20‐year follow‐up

Functional status was measured by the Glasgow Outcome Scale Extended (GOSE; Wilson, Pettigrew, & Teasdale, 1998). GOSE is based on a structured interview and provides an ordinal classification of disability into eight categories ranging from death to vegetative state, lower and upper levels of severe and moderate disability and lower and upper levels of good recovery.

Community integration was assessed by the Community Integration Questionnaire (CIQ; Willer, Ottenbacher, & Coad, 1994), a 15‐item scale to assess possible restrictions and effective role performance within three domains: the home integration (score ranges 0–10), social integration (score ranges 0–12) and productive activities (score ranges 0–7). Subscale scores in sum provide a total CIQ score ranging from 0 to 29. Higher scores indicate greater integration and fewer restrictions.

Health‐related quality of life (HRQL) was measured by the Medical Outcomes 36‐Item Short Form Health Survey (SF‐36; Ware, Gandek, & IQOLA Project Group, 1994). The SF‐36 measures HRQL along eight subscales: physical function (PF), role limitations due to physical health (RP), bodily pain (BP), general health (GH), vitality (VT), social function (SF), role limitations due to emotional health (RE) and mental health (MH). In addition, a single item reports the changes in overall health over the past year. Raw scores were transformed into a scale score ranging from 0 to 100 (worst to best). The subscales were calculated into the Physical Component Summary (PCS), consisting of the first four SF‐36 subscales (PF, RP, BP, and GH) and the MCS, consisting of the latter four SF‐36 subscales (VT, SF, RE, and MH).

Beck's Depression Inventory (BDI; Beck & Beamesderfer, 1974), a 21‐item self‐reported depression instrument was applied at the 10‐year follow‐up only. BDI score range is 0–63, with scores above 12 indicating depression.

### 2.2 Statistical analysis

Descriptive statistics were used to summarize the participants’ demographic and injury characteristics at baseline, as well as the demographics, and measures of function and HRQL at the 10‐ and 20‐years follow‐up. Wilcoxon signed‐rank test or paired *t*‐tests were used to assess the statistical differences between the measurements. The dependent variables in the two regression models were PCS and MCS at 20 years. Considering the small sample size, a conservative approach was applied using stepwise regression. With this method, the Statistical program selected which variables it would enter (stepping method criteria, probability of *F*, entry 0.05, removal 0.10) from a provided list of independent variables (age at injury and concurrent age, gender, GCS and associated injuries and 10‐year functional level (GOSE, CIQ and BDI scores). The PCS, MCS, and CIQ were modeled as interval variables while the measures of GOSE (severe/moderate disability vs. good recovery) and Beck Depression Inventory (BDI, 0–12 vs. >12) were modeled as binary.

The results were presented as adjusted *R* ^2^ and B coefficients (95% CI). The strongest models out of three were presented in Table 2. Prior to regression, multicollinearity, and model assumptions were examined using the tolerance and variance inflation factor (VIF). Distribution of the residuals was examined for normality, and influential data points were examined using Cook's distance.

Bar charts were presented to compare the percentage differences in GOSE levels, and mean differences in the CIQ and SF‐36 subscales between the 10‐ and 20‐years follow‐up. The mean measures of SF‐36 subscales at 10‐ and 20‐years follow‐up were further compared with the general population in Norway (Loge & Kaasa, 1998). All analyses were performed using the Statistical Package for the Social Sciences (SPSS, version 24). Statistical significance was set to *p* < 0.05.

## 3 RESULTS

Since the 10‐year follow‐up, 4 of the 62 patients had died. A total of 44 of 58 surviving patients (71% of the original cohort) consented to participation. Table 1 shows the main demographics and injury characteristics at baseline, 10‐ and 20‐year follow‐ups. Mean current age was 50.8 years (*SD* 11.4, range 36–75), and 73% were males. Patients with moderate and severe TBI were similar regarding age at the time of injury, gender, civil status, and education. At the 20‐year follow‐up, there were no differences between severity groups concerning age and civil status, whereas employment status differed significantly, as 75% of patients with moderate TBI were in full‐time jobs in contrast to 37% of patients with severe injury (*p* = 0.05).

**Table 1:** Demographics and injury characteristics at baseline, and functional status 10 and 20 years post‐TBI

	Moderate TBI, *n* = 20 (%)	Severe TBI, *n* = 24 (%)	*p*‐Value	Total sample, *N* = 44 (%)
Baseline data				
Gender				
Male	13 (65)	20 (80)	0.16	33 (75)
Female	7 (35)	4 (20)		11 (25)
Age (years), Mean (*SD*)	31.4 (12.3)	29.5 (10.2)	0.44	30.4 (11.2)
Civil status				
Married/cohabitant	11 (55)	12 (50)	0.51	23 (51)
Single	3 (15)	6 (25)		9 (21)
Living with parents	6 (30)	6 (25)		12 (28)
Employment				
Full‐time	15 (75)	22 (92)	0.39	37 (85)
Part‐time	1 (5)	0		1 (2)
Unemployed	3 (15)	1 (4)		4 (9)
Disability pension	1 (5)	1 (4)		2 (4)
Cause of injury				
Traffic accidents	13 (65)	17 (70)	0.68	30 (68)
Falls	5 (20)	5 (20)		10 (22)
Other	2 (15)	2 (10)		4 (10)
Isolated TBI	9 (45)	6 (25)	0.09	15 (34)
TBI with accompanying injuries	11 (55)	18 (75)		29 (56)
Intracranial surgery				
Yes	4 (20)	7 (32)	0.48	11 (25)
Length of acute hospital stay Median (range)	6 (2–15)	7.5 (2–64)	0.11	6 (2–64)
10‐year follow‐up				
Age (years), Mean (*SD*)	41.3 (12.5)	39.13 (10.3)	0.54	40.1 (11.3)
Civil status				
Married	14 (70)	14 (58)	0.27	28 (64)
Single	6 (30)	8 (32)		14 (32)
Living by parents	0	1 (5)		1 (2)
Missing	0	1 (5)		1 (2)
Employment				
Full‐time	15 (75)	6 (25)	*0.01*	21 (48)
Part‐time	2 (10)	6 (25)		8 (18)
Disability pension	3 (15)	12 (50)		15 (34)
GOSE levels				
3 (lower severe disability)	0	1 (4)	*0.01*	1 (2)
4 (upper severe disability)	0	2 (8)		2 (4)
5 (lower moderate disability)	0	8 (33)		8 (18)
6 (upper moderate disability)	4 (20)	7 (29)		11 (26)
7 (lower good recovery)	5 (25)	3 (13)		8 (18)
8 (upper good recovery)	11 (55)	3 (13)		14 (32)
CIQ total score, Mean (*SD*)	20.5 (3.8)	18.2 (4.6)	0.09	19.3 (4.5)
CIQ home integration score	5.9 (2.3)	6.1 (2.6)	0.80	6.0 (2.4)
CIQ social integration score	9.1 (2.1)	8.6 (2.3)	0.48	8.8 (2.2)
CIQ productivity score	5.5 (1.4)	3.7 (1.9)	*0.001*	4.5 (1.9)
SF‐36 scores, Mean (*SD*)				
Physical component summary (PCS)	76.0 (21.0)	67.0 (26.0)	0.16	71.0 (22.0)
Mental component summary (MCS)	79.0 (13.5)	71.0 (21.0)	0.32	75.0 (18.0)
BDI				
Depression (score >12)	4 (20)	5 (23)	0.83	9 (21)
No depression (score ≤12)	16 (80)	17 (77)		33 (79)
20‐year follow‐up				
Age (years), Mean (*SD*)	52 (10.6)	50.2 (12.4)	0.61	51 (11.3)
Civil status				
Married	14 (70)	14 (58)	0.65	28 (64)
Single	6 (30)	9 (37)		15 (34)
Living by parents	0	1 (5)		1 (2)
Employment				
Full‐time	15 (75)	9 (37)	*0.05*	24 (54)
Part‐time	0 (0)	2 (10)		2 (5)
Disability pension	3 (15)	9 (45)		12 (27)
Retired	2 (10)	4 (8)		6 (14)
GOSE levels				
3 (lower severe disability)	0 (0)	1 (4)	0.07	1 (2)
4 (upper severe disability)	0 (0)	1 (4)		1 (2)
5 (lower moderate disability)	2 (10)	9 (38)		11 (25)
6 (upper moderate disability)	4 (20)	4 (17)		8 (18)
7 (lower good recovery)	4 (20)	6 (25)		10 (23)
8 (upper good recovery)	10 (50)	3 (12)		13 (30)
CIQ total score, Mean (*SD*)	22.3 (3.2)	20.2 (3.3)	*0.04*	21.2 (3.4)
CIQ home integration score	7.6 (2.0)	6.2 (2.5)	0.06	6.8 (2.4)
CIQ social integration score	9.8 (1.8)	9.9 (1.8)	0.90	9.9 (1.5)
CIQ productivity score	4.9 (1.8)	4.3 (1.7)	0.23	4.6 (1.8)
SF‐36 scores, Mean (*SD*)				
Physical component summary (PCS)	83.0 (16.0)	77.0 (23.0)	0.16	80.0 (20.0)
Mental component summary (MCS)	80.0 (17.0)	71.0 (24.0)	0.32	75.0 (21.0)

*Notes*

BDI: Beck's Depression Inventory; CIQ: Community Integration Questionnaire; GOSE: Glasgow Outcome Scale Extended; SD: standard deviation; TBI: traumatic brain injury.

Italic values indicate statistically significant differences (*p* < 0.05).

### 3.1 Functional outcomes

#### 3.1.1 Glasgow Outcome Scale Extended

**Figure 1:**
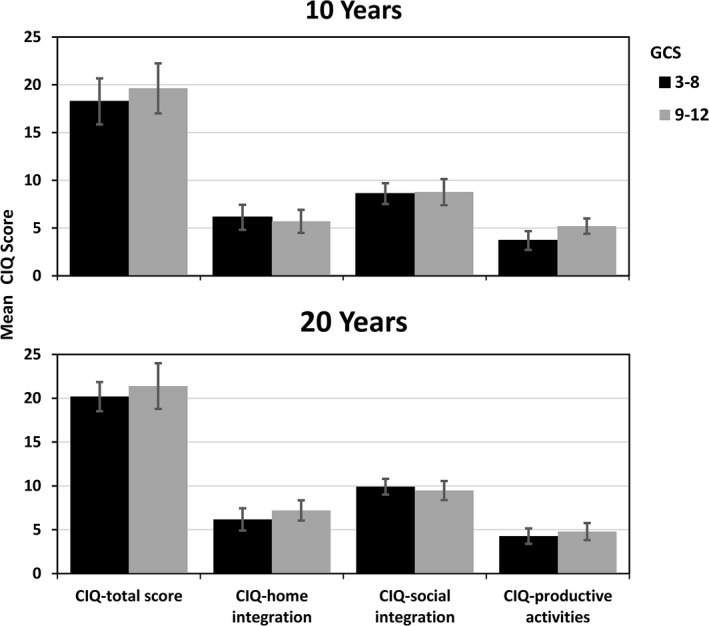
The distribution of GOSE levels in percentages at 10‐ and 20 years post‐TBI by injury severity groups

In the total sample, the median GOSE score at 20‐year follow‐up was 6.0 (Interquartile range, IQR 2.5). Twenty‐three patients (53%) had good recovery, while 19 (43%) presented with moderate, and two (4%) with severe disability, see Table 1. There were statistically significant differences in the GOSE median scores between injury severity groups (*p* = 0.003); 70% of patients with moderate TBI revealed good recovery, compared to 37.5% of patients with severe TBI, see Figure 1.

Regarding GOSE scores, there were no significant differences between males and females (*p* = 0.39), or between age groups dichotomized at the median value (<51 years vs. ≥51 years; *p* = 0.84). The overall GOSE median scores did not change significantly (*p* = 0.84) from 10‐ to 20‐year follow‐ups (medians 6.5 [IQR 2.5] and 7.0 [IQR 3]), respectively), and no significant differences in GOSE‐stability were found within the severity groups (moderate TBI: median 8.0 [IQR 1] vs. 7.5 (IQR 2.5), *p* = 0.26 and severe TBI: median 6.0 (IQR 2) vs. 6.0 [IQR 2], *p* = 0.14).

### 3.2 Community integration

The mean total CIQ score at 20‐year follow‐up was 21.2 (*SD* 3.4), with mean subscale CIQ scores being: Home Integration 6.85 (*SD* 2.4); Social Integration 9.9 (*SD* 1.5); and Productive Activity 4.6 (*SD* 1.8).

**Figure 2:**
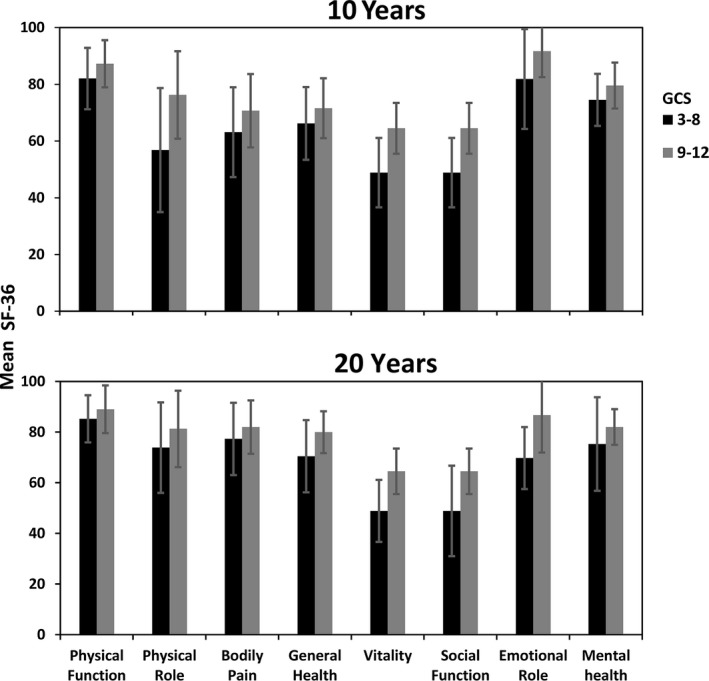
Community Integration Questionnaire (CIQ) Mean scores 10 and 20 years post‐TBI by injury severity groups

There were no significant differences effects of gender (*p* = 0.69), nor age (*p* = 0.38) in the total CIQ mean scores. There was a statistically significant difference in the total CIQ mean scores between patients with moderate and severe TBI (*t* = −1.94, *p* = 0.04), but not for the CIQ subscales (see Figure 2).

A statistically significant improvement was found on the total CIQ mean scores at the 20‐years follow‐up compared to 10 years (means 21.2, *SD* 3.4 vs. 19.4, *SD* 4.5, respectively, *p* = 0.01; see Figure 2). Of the CIQ subscales, the Social Integration showed statistically significant improvements from 10 to 20 years (means 8.8 *SD* 2.2 vs. 9.9 *SD* 1.5, respectively, *p* = 0.005). Mean scores changes within the severity groups from 10‐ to 20‐year follow‐up showed statistically significant differences for total CIQ mean score (moderate TBI *p* = 0.02, severe TBI *p* = 0.08), Home Integration (moderate TBI *p* = 0.002) and Social Integration (severe TBI *p* = 0.004). Productive Activity mean scores approached significance in both severity groups (*p* = 0.08).

### 3.3 Health‐related quality of life

**Figure 3:**
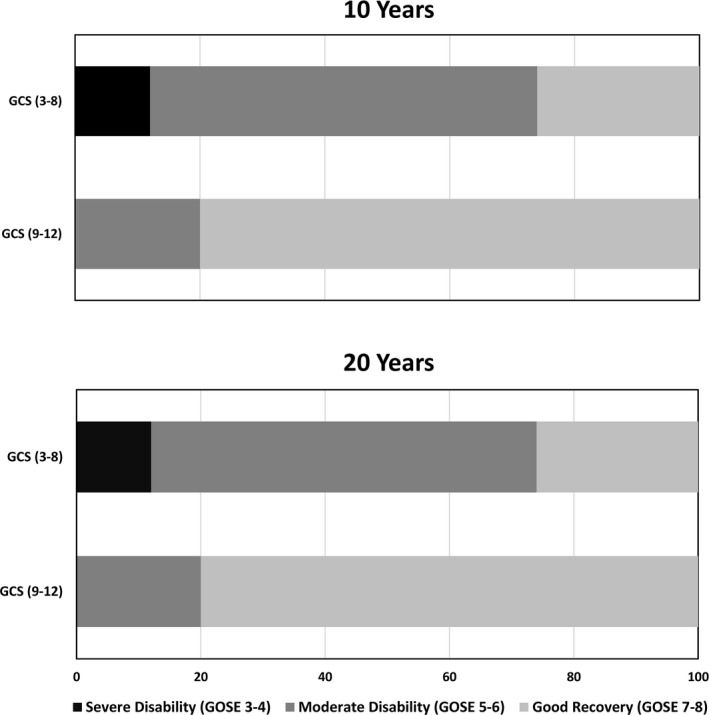
SF‐36—Mean scores 10 and 20 years post‐TBI by injury severity groups

The mean score of the SF‐36 domains is displayed in Figure 3. There were no significant differences in the mean scores of any SF‐36 dimensions between males and females, age groups or TBI severity.

There were no significant differences in the mean scores of SF‐36 subscales between 10 and 20 years, except for Bodily Pain, with higher scores at 20 years indicating less pain (67.4 vs. 80.4 *p* = 0.02, respectively). Role Emotional mean score approached significance (*p* = 0.06). The analysis of changes in mean scores within the severity groups from 10 to 20‐year follow‐up showed statistically significant differences for: General Health (moderate TBI 71.6 vs. 80.0 *p* = 0.03, indicating better general health at 20‐years), and approached significance for Bodily Pain (severe TBI 64.2 vs. 79.0 *p* = 0.08 indicating less pain) and Role Emotional (severe TBI 85.7 vs. 71.5 *p* = 0.09 indicating a trend toward worsening).

### 3.4 Factors associated with PCS and MCS 20‐years after injury

The multiple regression models are presented in Table 2. The adjusted *R* ^2^ of PCS was 29%, meaning that the predictors (BDI and CIQ productivity score at 10‐years) explained almost one‐third of the PCS variance at 20‐years. The B coefficient was negative for BDI meaning that depression at 10‐years predicted poorer PCS 20‐years after TBI. Furthermore, the B coefficient was positive for CIQ productivity, implying that productive activities at 10‐years predicted better PCS at 20‐year.

**Table 2:** Results from the multiple regression models of SF‐36 component summary scores (*n* = 42)

Variables	MCS 20‐year		PCS 20‐year	
	B coefficient (95% CI)	*p*‐Value	B coefficient (95% CI)	*p*‐Value
Constant	78.64 (58.3; 98.9)	<0.001	68.67 (53.59; 83.74)	<0.001
Gender	−14.77 (−26.28; −3.6)	0.013		
BDI 10‐year	−16.45 (−29.5; −3.4)	0.015	−20.63 (−38.45; −6.78)	0.005
CIQ productivity 10‐year	4.9 (1.53; 6.85)	0.003	3.39 (0.5; 6.38)	0.023

*Notes*

Adjusted *R* ^2^: MCS = 0.446; PCS = 0.294.

BDI: Beck's Depression Inventory; CIQ: Community Integration Questionnaire; MCS: mental component summary; PCS: physical component summary.

The adjusted *R* ^2^ of MCS was 45%, meaning that factors such as gender, BDI and CIQ productivity scores at 10‐years explained almost half of the variance in MCS at 20‐years. The B coefficients were negative for gender and BDI meaning that female gender and depression at 10‐years predicted poorer MCS at 20‐years, whereas the B coefficient was positive for CIQ productivity, meaning that productive activities at 10‐years predicted better MCS at 20‐years.

### 3.5 HRQL comparison with the general population of Norway

**Figure 4:**
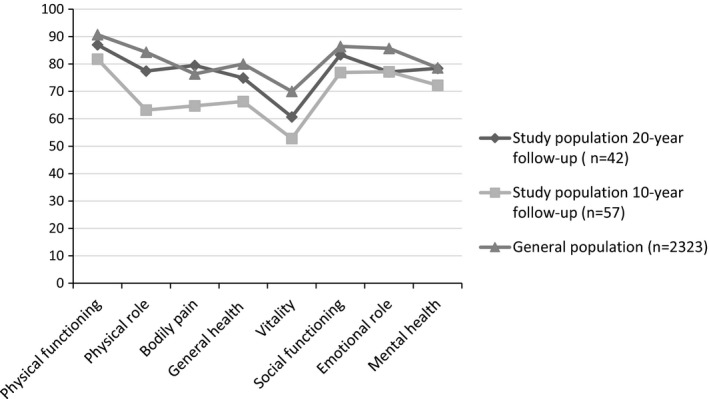
Mean SF‐36 subscale score profile; the study population (*n* = 42) at 20 year postinjury compared to the study population (*n* = 57) at 10 year postinjury and general population of Norway (*n* = 2,323)

Figure 4 presents the mean SF‐36 scores 10 and 20 years postinjury compared to the Norwegian general population (Loge & Kaasa, 1998). The SF‐36 subscales are presented from left to right; the four‐first subscales cover physical health and the four last subscales cover mental health. Twenty years post‐TBI, the SF‐36 mean scores were comparable with the general population, except for Emotional role (with score difference >5 scale points).

## 4 DISCUSSION

This study described disability levels and HRQL 20 years after moderate and severe TBI, changes in global functioning, community integration and HRQL from 10 to 20 years, and factors associated with physical and mental health.

In terms of demographic variables and TBI severity, differences were found only in employment status at 20‐years post injury, where 75% of patients with moderate TBI were in full‐time jobs compared to 37% with severe TBI. Employment rates remained stable in the moderate TBI group from baseline to 10‐ and 20‐year follow‐up. The total percentage of working patients in the severe TBI group remained unchanged from 10 to 20 years follow‐up, and is similar to numbers reported by Wood (2008). However, the number of patients with severe TBI working full‐time is 12% higher at the 20‐year follow‐up compared to the 10‐year follow‐up, suggesting a long‐term potential for increased work‐participation, particularly in those working part‐time. Nonetheless, the percentage of patients with severe TBI receiving disability pension remained unchanged. As the patients with severe TBI included in this study are still in their productive ages, a loss of work productivity results in a substantial financial burden both at the individual and societal levels (Tuominen, Joelsson, & Tenovuo, 2012). This highlights the importance of providing vocational rehabilitation services and vocational support to optimize community integration and work participation also in the very long‐term perspective post‐TBI (Brown et al., 2011).

Global functional status was stable from 10 to 20 years, except for a tendency toward slight improvement in patients with severe TBI. Most patients showed good recovery (53%) or moderate disability (43%), as measured by GOSE at 20 years of follow‐up. However, a very small sample of participants (4%) had severe disability. These values are consistent with the findings of previous studies (McMillan et al., 2012; Schulz‐Heik et al., 2016). Roughly two‐thirds of the patients with moderate TBI revealed good recovery in contrast to one‐third of patients with severe TBI indicating a long‐term differential effect of severe and moderate TBI which is in line with findings from previous research (Forslund et al., 2017; Ponsford et al., 2008).

The overall community integration at 20‐year follow‐up did not differ from the general population (Corrigan, 1994), suggesting successful long‐term community reintegration (Wood & Rutterford, 2006). Age and gender had no impact on community reintegration. However, injury severity influenced community integration, in line with previous studies (Andelic et al., 2015; Sandhaug, Andelic, Langhammer, & Mygland, 2015; Winkler, Unsworth, & Sloan, 2006). The results also showed significant improvements in the community integration from 10 to 20 years both in between and within TBI severity groups. The Social Integration subscale showed the greatest change in the total sample and in patients with severe TBI, whereas Home Integration was greater in patients with moderate TBI. These results may suggest that these domains of community integration can improve in a long‐term perspective; in line with the study by Brown et al. (2011) which indicated that adaptation to impairment‐related limitations improves as the time since injury increases.

In the evaluation of HRQL at the 20‐year follow‐up, no statistically significant differences were found between SF‐36 dimensions and age, gender or injury severity groups. The latter is in line with results from the previously mentioned studies by Jacobsson and Nestvold (Jacobsson et al., 2010; Nestvold & Stavem, 2009). With respect to the 10‐ to 20‐year follow‐up, reduction in bodily pain from 10 to 20 years may suggest that the physical TBI‐related consequences are weakened over time, as reported in other studies (Soberg, Bautz‐Holter, Finset, Roise, & Andelic, 2015). Furthermore, individuals with TBI may adapt over time and experience a large degree of psychological and emotional normalization and better HRQL in the long‐term perspective (Bonanno, 2004; Brown et al., 2011).

Factors such as depression and productive activities in the first decade after injury may influence subsequent physical health, as depression and CIQ productivity at 10 years explained one‐third of the variance in PCS at the 20‐years follow‐up. These predictors, along with female gender, accounted for almost half of the variance in MCS at 20 years. In this sense, the presence of depression at 10‐year follow‐up, together with being female, influenced mental health negatively at 20 years of follow‐up. Previous studies on TBI (McCarthy et al., 2006), and the general population have also reported more psychosocial distress and worse mental health in females as compared to males (Loge & Kaasa, 1998). The results of the influence of depression are in line with other studies indicating that depression is strongly associated with HRQL (Steadman‐Pare et al., 2001). In contrast, CIQ productivity at 10 years influenced physical and mental health positively 20 years after the injury. This is also line with previous studies that have reported that productivity is a cornerstone to achieving a good HRQL (Jacobsson et al., 2010) through greater self‐fulfillment and increased opportunities.

Taken together, the study results which included a multidimensional evaluation of functioning 20‐years postinjury are clinically relevant and may provide a basis for the development of long‐term targeted follow‐up programs, and specific strategies for healthcare service delivery and goals of professional assistance, in order to promote positive outcomes over the life span after TBI.

This study has several limitations that should be addressed. First, all study participants initially included were individuals with moderate and severe TBI, between 16 and 55 years old, and received care in the South‐Eastern region of Norway. This may limit the generalizability to other populations. In addition, the statistical analyses were limited by a small sample size. Attrition to follow‐up is a common problem for any long‐term longitudinal study, and this may introduce selection bias, as the most improved patients often remain available (Gray et al., 2017). In this study, 70% of the original cohort consented for 20‐year follow‐up, which we consider acceptable. Only 4% had severe disability on the GOSE, and 53% showed good outcome. We can therefore not rule out that the sample may have been biased toward individuals who survived from moderate‐to‐severe TBI and are living in the community, and those who were able to self‐report their outcomes.

In conclusion, the rate of disability reported in the study at 10 years remains stable at 20 years after injury, while the community integration as well as the HRQL has reached values similar to the general populations. The fact that levels of disability remained unchanged, calls attention to the human capacity to adapt over time, and to obtain good community integration and quality of life despite continued and chronic consequences of TBI. Female gender and depression influenced the health outcomes negatively, whereas productivity was an important positive factor for better physical and mental health. The findings have important implications for management of patients in the chronic phase of TBI indicating the need for long‐term tailored follow‐up programs to allow detection and intervention for depression and other TBI‐related health problems, and training in self‐management to improve health and well‐being. Furthermore, access to comprehensive and coordinated rehabilitation services and vocational support in the very long‐term post‐TBI to optimize community integration and participation in productive activities, and to educate patients and their families to prevent or reduce late‐developing problems is important. This is in line with a comprehensive review regarding brain injury rehabilitation, showing that comprehensive and holistic rehabilitation can improve community integration, functional independence, and productivity, even for patients who are many years post injury (Cicerone et al., 2011).

## CONFLICT OF INTEREST

The authors report no conflict of interest.
