# CORRIGENDUM

**DOI:** 10.1002/brb3.2120

**Published:** 2021-06-16

The article by Andelic et al. (2018) was published with incorrect figure placements and figure captions. Figure 3 should be Figure 1, Figure 1 should be Figure 2, and Figure 2 should be Figure 3. Corrected figures appear below (Figures 1, 2, 3).

**Figure 1:**
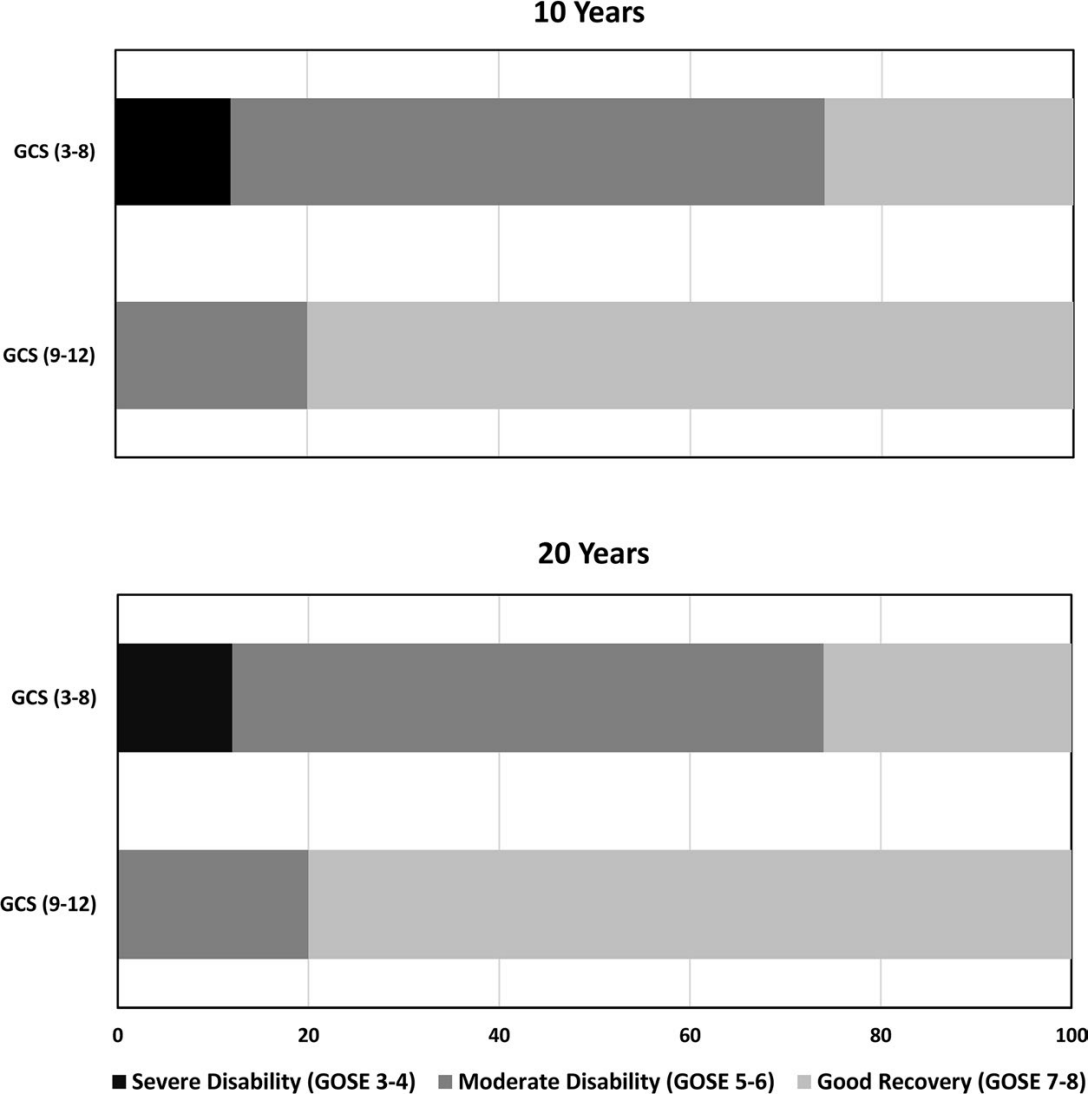
The distribution of GOSE levels in percentages at 10‐ and 20 years post‐TBI by injury severity groups

**Figure 2:**
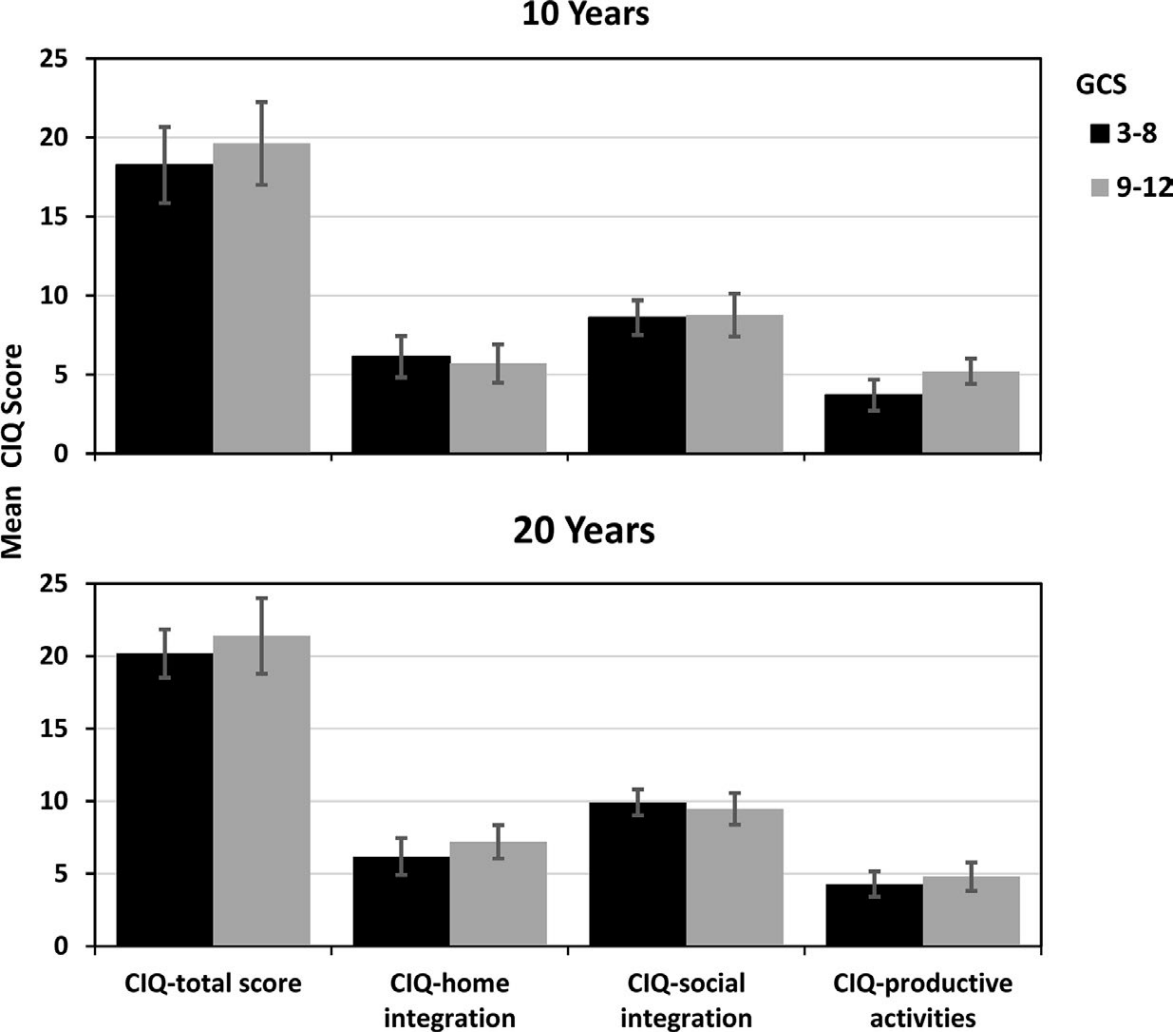
Community Integration Questionnaire (CIQ) Mean scores 10 and 20 years post‐TBI by injury severity groups

**Figure 3:**
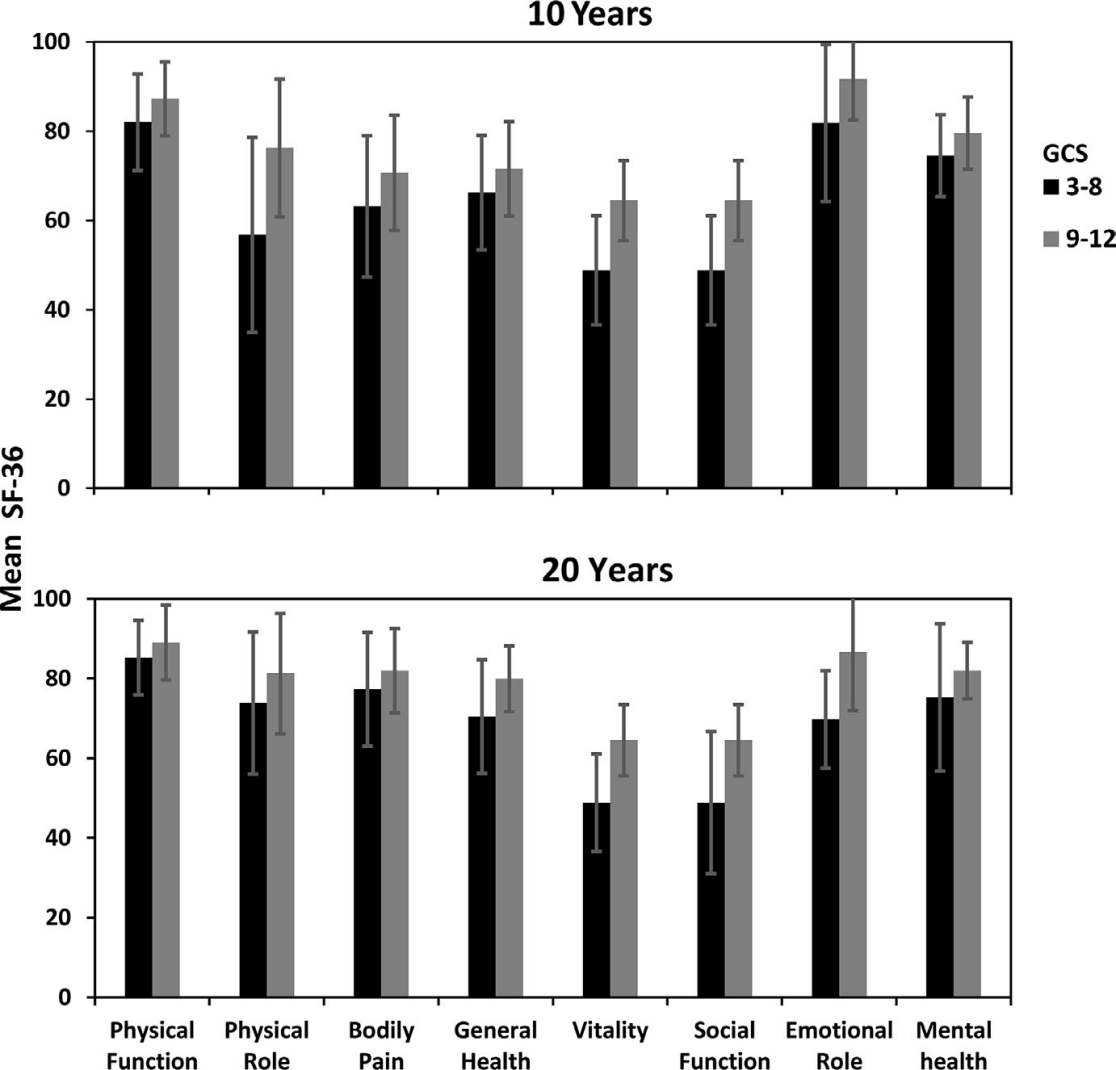
SF‐36—Mean scores 10 and 20 years post‐TBI by injury severity groups

The authors regret the error.
